# Technical Assistance to Enhance Prevention Capacity: a Research Synthesis of the Evidence Base

**DOI:** 10.1007/s11121-016-0636-5

**Published:** 2016-02-09

**Authors:** Jason Katz, Abraham Wandersman

**Affiliations:** Domestic Capacity Building Service Area, American Institutes for Research, 1000 Thomas Jefferson St., Washington, D.C., NW 20007 USA; Department of Psychology, University of South Carolina, 1512 Pendleton Street, Columbia, SC 29208 USA

**Keywords:** Technical assistance, Implementation science, Dissemination

## Abstract

Despite the availability of many evidence-based prevention interventions (EBIs), gaps exist in bringing these programs into widespread practice. Technical assistance (TA) is a strategy for enhancing the readiness of practitioners to implement EBIs. Although many millions of dollars are spent on TA each year, there is little consensus about what the essential features of TA are and how to provide TA with quality. A broad-based research synthesis methodology was used for analyzing the current evidence base for TA using three frames: (1) applying the Getting To Outcomes (GTO) model for categorizing evidence on TA that specifies tasks for planning, implementing, and evaluating TA; (2) understanding the relevance of a successful relationship between the TA provider and TA recipient; and (3) considering the extent to which TA fits the life cycle needs of the preventive intervention. Results indicated that an explicit model or organizing framework is rarely used to plan, implement, and/or evaluate TA; specific TA tasks performed vary widely across studies; TA is rarely delivered to recipients who are seeking to sustain innovations subsequent to adoption and implementation; however, there is systematic attention to relationships and relationship-building. Overall, this synthesis indicates that the extent to which TA is being delivered systematically is limited. We suggest that funders and other stakeholders develop and implement standards for TA quality in order to ensure that many of these limitations are addressed.

**Electronic supplementary material:**

The online version of this article (doi:10.1007/s11121-016-0636-5) contains supplementary material, which is available to authorized users.

## Introduction

The Institute of Medicine’s (IOM) 2015 report, *Unleashing the Power of Prevention*, calls for “reducing the incidence and prevalence of behavioral health problems in the population of young people from birth through age 24 by 20% over the next decade” (Hawkins et al. 2015, pg. 12) by having practitioners use evidence-based prevention programs. The report recognizes the need for coaching and technical assistance (TA) to bridge the gap between research and practice. This article is based on the premise, described by Wandersman et al. (2012), that if it is important to be evidence-based about our interventions, then it is important to be evidence-based about the support provided to practitioners via tools, training, and TA. In this article, we focus on the evidence base for TA using three frames: tasks, relationships, and connections to the life cycle stage of an innovation. Innovations are defined as programs, policies, or practices that are new to a setting.

## The Interactive Systems Framework for Dissemination and Implementation

Practitioners are often slow to adopt and use evidence-based practices with quality. One strategy to help ameliorate this gap is to develop and test interventions in the same or similar contexts in which they will be used (e.g., Brownson et al. 2013; Klesges et al. 2005; Neta et al. 2015). Another important strategy is to provide TA, as described in the Interactive Systems Framework (ISF) for Dissemination and Implementation (Wandersman et al. 2008). The ISF was developed with the Centers for Disease Control and Prevention to address the gap between science and practice using three interactive systems: (1) synthesis and translation system, (2) delivery system, and (3) support system. The ISF’s synthesis and translation system extends the products of research into user-friendly formats that can be accessed with ease and understood by practitioners in the ISF’s delivery system. The ISF’s delivery systems are the organizations or community settings that implement interventions to reach their desired prevention and health promotion outcomes. The support system involves intermediary organizations that provide support (e.g., via training and TA) to bring the information from the synthesis and translation system into practice with quality by the delivery system (diagram of the ISF is available online).

## Conceptualization of TA

TA is an individualized and hands-on approach to capacity-building in organizations and communities, often conducted after training (Chinman et al. 2005; Keener 2007). Each year, many millions of dollars are spent on TA to help service delivery systems implement innovations with quality. To provide just two examples of TA centers, the U.S. Department of Health and Human Services’ Substance Abuse and Mental Health Services Administration and the U.S. Department of Education’s Office of Safe and Healthy Students both fund national TA centers to support states and local entities in creating safe and healthy school environments in which students can learn and thrive. However, in spite of all the money that is spent and the importance of the issues being addressed, there is an apparent absence of a model or organizing approach for how to deliver TA with quality. Similar to the early days of psychotherapy, little is known about what actually happens (or should happen) in the TA process. The field of TA requires an operationalization of TA that defines the TA process into specific steps or components, which can be replicated across TA projects (with appropriate customizations) and studied empirically to develop an evidence base for conducting TA with quality to achieve results (Wandersman et al. 2012).

Much more research is needed to identify what the functional components of TA are (Fixsen et al. 2005). Some research suggests the importance of considering the developmental stage of the recipient to inform how TA should be delivered, including the appropriate dosage of TA (e.g., “intensive TA” vs. “basic TA”) and mode of delivery (on-site vs. virtual) (Blase 2009; Feinberg et al. 2008). Other articles have emphasized the need to provide TA that targets multiple kinds of capacity, including provider competencies as well as organization or system changes (O’Donnell 2009; Winton and Catlett 2009). There appears to be little consensus about what the essential features of TA are, and how to provide TA with quality. In this article, we describe a research synthesis performed to identify how TA is being delivered in prevention.

To guide the synthesis, we identified three frames for extracting information from the literature about TA: tasks, relationships, and the innovation life cycle. Tasks are processes or methods that are used to plan, implement, and evaluate TA. Relationships refer to human encounters between TA providers and recipients, including factors such as trust and respect. The innovation life cycle refers to the stage of evolution of an innovation being implemented in a delivery system, and the life cycle-related TA needs of the innovation that is being supported. These three frames were operationalized in the following ways:

## Tasks

In relation to TA in the ISF, a TA provider works with the delivery system—as illustrated in the ISF figure by an arrow connecting the delivery system and support system. For example, when TA providers are working with an organization, they could perform a needs and resources assessment of the organization to see which capacities are strong and which capacities the TA provider and the organization decide that the TA provider should help them build, i.e., the TA provider is doing a needs and resources assessment in deciding which priorities to work on with the organization. (Note that this is different from supporting the organization to help it conduct a needs assessment of the community.) Key issues in TA are as follows: what tasks does the TA provider do with the delivery system, and how frequently are they performed? As described in *Toward an evidence-based system for innovation support*… (Wandersman et al. 2012), it is important to have a conceptual frame for organizing an evidence base on support. We chose Getting To Outcomes® (GTO®)^1^ in this research synthesis as a planning, implementation, evaluation, and continuous quality improvement frame. GTO is the frame used in Wandersman et al.’s (2012) conceptualization of an evidence-based system of support. GTO consists of ten steps for planning, implementing, and evaluating with quality (see Table 1). There are tasks associated with each of the ten GTO steps. For example, Step 3 (best TA practices) involves reviewing the best available evidence to select practices that can be used to reach goals. GTO is widely used. GTO manuals on the RAND website have been downloaded over 110,000 times, and several federal agencies have required community and organizational grantees to use it to implement evidence-based interventions (e.g., Office of Adolescent Health). GTO is also being used in a SAMHSA-funded training and TA center coordinated by the American Institutes for Research to guide TA providers to provide TA in an accountable evidence-informed way.

**Table 1:** Overview of frames for coding TA articles

Frames	Description
Tasks	
TA needs and resources (Step 1)	What recipient assets can be applied to the initiative? What general capacities, and what innovation-specific capacities, should TA help the recipient to build?
TA goals and desired outcomes (Step 2)	What specific outcomes should the TA be designed to achieve, based on #1?
Best TA practices (Step 3)	What best TA practices (e.g., practices based on a strong theory or rationale) can be used to reach the TA outcomes?
Fit (Step 4)	Do the best TA practices appropriately match the recipient’s circumstances?
Capacity (Step 5)	Are there sufficient capacities (e.g., time, technology, manpower, partners, funds) to put the best TA practices into action?
Planning (Step 6)	What is the plan for implementing the selected TA practices?
Process evaluation (Step 7)	To what extent is the TA plan being implemented with quality?
Outcome evaluation (Step 8)	Have the desired TA outcomes been accomplished?
Continuous quality improvement (Step 9)	What continuous quality improvement strategies are being used to improve TA over time?
Sustainability (Step 10)	When TA outcomes are accomplished, how can they be sustained over time?
Relationship features	
Trust	TA recipient’s faith or confidence in the TA provider.
Respect	Quality or state of being esteemed (holding in high regard).
Collaboration	TA providers and recipients work together in the direction of a shared purpose.
Adjusting to readiness	TA provider structures the TA process to match the recipient’s perception of how important change was at that moment.
Strengths-based	TA provider focuses on current assets and/or inspires the recipient with courage or hope.
Autonomy-supportive	TA provider promotes self-governance on the part of the TA recipient.
Rapport	Collegiality and/or a cooperative interpersonal climate.
Life cycle stages	
Initiation	Primary focus on general capacities in the delivery system, including leadership, availability of resources needed for implementation, work climate, and staffing.
Implementation	Primary focus on the active work involved in implementing a specific innovation, including logistics and planning, and using skills and expertise for successful implementation.
Stability	Primary focus on sustaining an innovation within the organization or system.

Articles in this research synthesis were reviewed to identify which models/organizing approaches (including GTO) were used to guide TA provision, and, more specifically, which TA tasks associated with each of the ten GTO steps were reported. Research questions for this frame were as follows: which (if any) models, frames or organizing approaches for TA are reported in the literature? Based on the GTO-TA frame, which tasks were conducted in TA—e.g., strategic planning (including needs and resource assessment, goal setting, selection of best TA practices, assessment of fit, and capacity of best practices), implementation/process evaluation, outcome evaluation of TA, continuous quality improvement, and sustainability?

## Relationships

A good relationship between the TA provider and TA recipient is important (Mitchell et al. 2002; Wandersman et al. 2012). We were not able to find a pre-existing model that specified important aspects of the TA relationship. Therefore, we developed a list of relationship features reported in an initial sample of articles in closely related bodies of literature (implementation coaching, consultation, adult mentorship) and used this list to code the literature (see Table 1). The research question for this frame was as follows: which features of a quality TA relationship are reported in articles?

## Innovation Life Cycle

We adapted a model (Urban et al. 2014) that describes the stages that a delivery system typically passes through when implementing an innovation (see Table 1). We assessed differences in tasks and relationships at each of the stages. The research question for this frame was as follows: are there differences in TA tasks and relationships across different life cycle stages of an innovation?

## Methods

A broad-based research synthesis (Labin 2008) was conducted to analyze the TA literature. The research synthesis method shares some of the core features and underlying logic of a meta-analysis, yet has the added advantage of being able to accommodate an array of project designs (including case studies, descriptive studies, and quasi-experimental designs). The methodology overlaps with PRISMA requirements (Moher et al. 2009), with six steps, summarized below.

1. Defining the research questions (see above)
2. Collecting information sources
  Four literature search engines were utilized to identify peer-reviewed articles for review: MEDLINE, PsycInfo, CINAHL, and Social Work Abstracts. The search terminology used was “technical assistance and (evaluation or outcomes).” The rationale for this search terminology was to allow for a large initial set of articles about TA that have an empirical basis. No time restrictions for published articles were used (articles used in the synthesis ranged from 1986–2013), and articles were necessarily written in English. As indicated in supplementary materials detailing the coding process (available online), over 800 articles were initially identified.
3. Selecting information sources based on inclusion criteria
  Original articles (not reviews, commentaries, etc.) in peer-reviewed journals were accepted. Studies included quantitative and/or qualitative methods. Articles that were accepted needed to be consistent with the conceptualization of TA as an individualized and hands-on approach to capacity-building in organizations and communities. To ensure that TA was consistent with how it is understood in the three boxes of the ISF (delivery system, support system, and synthesis and translation system), information sources that addressed TA needed to satisfy two additional conditions in order to be included in the synthesis: (1) TA needed to occur within the context of dissemination and implementation projects (e.g., prevention projects that were funded by departments in the federal government, foundations, or state or local governments) and (2) TA was necessarily delivered via a formal and explicit support system that was external to the organization (e.g., mentoring from within the organization would not be included). As indicated in supplementary materials detailing the coding process (available online), 111 articles met the inclusion criteria and were coded for this synthesis.
4. Extracting and coding data
  Using a structured coding form (available online, with an accompanying codebook), each article that met the inclusion criteria was coded according to the major elements in the three synthesis frames. The unit of analysis for coding was the article and not the project being described (there were not any cases in which multiple articles described the same project, with the exception of several articles that described different time periods or aspects of large projects). An inter-rater reliability analysis was conducted with a set of 12 articles. A Ph.D. level volunteer collaborated on this work, independently rating the 12 articles. Prior to the inter-rater reliability analysis, there were several rounds of practice coding with discussions and consensus-building. The calculated percentage agreement for the inter-rater reliability analysis was 87 %. However, to account for the possibility of chance agreement, Cohen’s kappa statistic was also computed, which provides a more conservative estimate of agreement (Landis and Koch 1977). The kappa was 0.73 (indicating substantial agreement). Therefore, methods for the literature review involved coding by a single evaluator, with a mid-course booster to prevent drift (Labin et al. 2012).
5. Analyzing data
  Descriptive and inferential analyses were conducted. Descriptive analyses involved looking at the total information sources as the denominator and a relevant indicator (corresponding to major research questions in this synthesis) as the numerator. A sample question is “number of information sources that address needs/resources assessment/total number of information sources.” More specific sub-questions were also included, such as “number of information sources that specify the use of surveys/total number of information sources that address needs/resources assessment.” Inferential analyses were conducted to compare tasks and relationships at different innovation life cycle stages, using the chi square test of independence, or Fisher’s exact test when one of the cells in a cross tabulation table had an expected frequency of five or less (Anderson 2011).
6. Presenting findings
  Information about the use of tasks according to the ten GTO steps is initially presented, followed by information about TA relationship features (e.g., trust, collaboration). Finally, information is provided about the life cycle stages (initiation, implementation, stability) in which the delivery of TA is contextualized, and comparisons of the stages in terms of tasks and relationship features.

## Results

Of the articles reviewed, 87 addressed prevention (78 %) (e.g., prevention of chronic illness), 17 treatment (15 %) (e.g., treatment of serious mental illness), and 7 were ambiguous (6 %). Articles about prevention focused on an array of behavioral and health outcomes, including worker safety in factories, smoking, and teen pregnancy (see pie chart available online). Chi square analyses were conducted to test for possible differences between articles coded as prevention vs. treatment with respect to a sample of major items in each of the frames; no significant differences were identified.

TA mode (on-site vs. off-site/virtual) and dose (amount of TA provided in terms of time or number of sessions) were reported in most articles. Both on-site TA (68/111 = 61.3 %) and virtual TA (61/111 = 55.0 %) were highly utilized, and 74 articles explicitly reported dosage (66.7 %). A majority of the TA services were described as being ongoing or involving regularly scheduled activities (69/111 = 62.2 %), which in some cases was reported as “proactive,” while considerably fewer articles involved recipient response-driven TA (27/111 = 24.3 %).

## TA Tasks

### Which (If Any) Models, Frames, or Organizing Approaches for TA Are Reported in the Literature?

Only two articles specified models or organizing approaches for planning, implementing, and/or evaluating TA tasks. The first of these was a four-phase TA process (assessment, cooperative planning, delivery of TA, and evaluation) (Nemec et al. 1991). For example, the assessment phase involved a focus on the readiness of the recipient, including the extent to which the recipient’s organizational culture facilitated implementation of the innovation, and the value that the recipient ascribed to TA in terms of the assistance and supports being offered. The assessment phase also focused on the resources that are expected to help the recipient to receive TA (e.g., supportive leadership). The second model was not very specific; it broadly mentioned a three-phase approach involving initial diagnosis, development of a logic model, followed by a planning process around how the TA services will make improvements (Chinman et al. 2013). No other articles indicated a model or organizing approach with a sequence of tasks guiding the TA process.

### Which Tasks Are Used for TA Strategic Planning, Implementation, Evaluation, and Sustainability?

Table 2 shows how the tasks proposed in GTO steps were mentioned in the reviewed articles. The most heavily reported steps were TA needs/resource assessment (GTO Step 1) (73/111 = 66 %), setting TA goals (GTO Step 2) (97/111 = 87.4 %), conducting process evaluation (GTO Step 7) (56/111 = 50.5 %), and conducting outcome evaluation (GTO Step 8) (87/111 = 78.4 %). A smaller percentage of articles (<50 %) reported tasks associated with best TA practices (GTO Step 3) (44 /111 = 39.6 %), TA planning (GTO Step 6) (10/111 = 9 %), continuous quality improvement (GTO Step 9) (13/111 = 11.7 %), and sustainability (GTO Step 10) (31/111 = 27.9 %).

**Table 2:** TA tasks, relationship features, and life cycle stages reported in articles

Frames	Frequency (%)
Tasks	
TA needs and resource assessment (GTO Step 1)	73/111 (66)
Needs/resource data collection process	69/111^a^ (62.2)
Survey	40/111 (36.0)
Interview	15/111 (13.5)
Focus group	4/111 (3.6)
Reporting a timeline to guide data collection	41/111 (36.9)
Data analysis process	55/111 (49.5)
Reporting of results	60/111 (54.1)
Interpretation of results	40/111 (36.0)
Setting TA goals (GTO Step 2)	97/111 (87.4)
Setting goals based on needs and resources assessment	5/111 (4.5)
Translating goals into desired outcomes	7/111 (6.3)
Benchmarking	3/111 (2.7)
Best TA practices (GTO Step 3)	41/111^b^ (39.6)
Diffusion of innovation-oriented tasks	7/111 (6.3)
Adult learning tasks	5/111 (4.5)
Academic detailing tasks	2/111 (1.8)
Participation/empowerment tasks	11/111 (9.9)
Other step 3 tasks	13/111 (11.7)
Fit of best TA practices (GTO Step 4)^e^	25/41 (61)
Fit with recipient’s readiness to receive TA	5/41 (12.2)
Fit with recipient’s daily activities and organizational operations	11/41 (26.8)
Fit with recipient’s organizational culture	5/41 (12.2)
Fit with recipient’s other priorities, timelines, and/or deliverables	5/41 (12.2)
Fit with recipient’s other existing support	1/41 (2.4)
Capacity to implement best TA practices (GTO Step 5)	32/41 (78)
Human capacity	32/41 (78.0)
Fiscal capacity	10/41 (24.4)
Technical capacity	1/41 (2.4)
Planning for TA delivery (GTO Step 6)	10/111 (9)
Using a collaborative TA planning process	5/111 (4.5)
Setting a timeline for TA delivery	1/111 (0.9)
Establishing roles and responsibilities pertaining to TA delivery	1/111 (0.9)
Process evaluation of TA delivery (GTO Step 7)	56/111 (50.5)
Assessment of quality	7/111 (6.3)
Assessment of reach	28/111 (25.2)
Assessment of dosage	26/111 (23.4)
Assessment of satisfaction	28/111 (25.2)
Making midcourse corrections	5/111 (4.5)
Outcome evaluation (GTO Step 8)	87/111 (78.4)
Outcome evaluation data collection process	36/111 (32.4)
Survey	26/111 (23.4)
Interview	17/111 (15.3)
Focus group	4/111 (3.6)
Consistency with Step 2 goals	70/71^c^ (98.6)
Reporting of results	87/111 (78.4)
Continuous quality improvement (GTO Step 9)	13/111^b^ (11.7)
Tasks for continuous feedback	1/111 (0.9)
Quality improvement consortia/communities of practice	0/111 (0.0)
Plan-do-study-act process	6/111 (5.4)
Sustainability (Step 10)	31/111^b^ (27.9)
Sustainability plan	0/111 (0.0)
Selection of a champion	4/111 (3.6)
Integration of TA into delivery system	13/111 (11.7)
Relationship features	
Relationships addressed	52/111 (46.8)
Trust	8/111^d^ (7.2)
Respect	4/111 (3.6)
Collaboration	28/111 (25.2)
Adjusting to readiness	2/111 (1.8)
Strengths-based	15/111 (13.5)
Roles and responsibilities	0/111 (0.0)
Autonomy-supportive	8/111 (7.2)
Rapport	4/111 (3.6)
Other relationship features	17/111 (15.3)
Life cycle stages	
Initiation of an innovation	47/111 (42.3)
Implementation of an innovation	55/111 (49.5)
Stability of an innovation	7/111 (6.3)
Information N/A about life cycle stage	2/111 (1.8)

^a^Articles may be repeated within subheadings for steps reported in this table

^b^A subset of these articles mentioned the step generally without pausing to specify what the specific tasks were

^c^Denominator reflects number of articles that report Step 2 goals

^d^Multiple relationship features can be addressed in an article

^e^Since Steps 3, 4, and 5 go together, the number of articles addressing best TA practices was used as the denominator for Steps 4 and 5

Analyses of activities occurring within steps indicated substantial variability. Articles specified a relatively large number of needs/resource assessment (Step 1) activities. For example, 62 % specified a data collection process (including use of surveys, focus groups, and key informant interviews). Although 87 % of articles reported setting general TA goals (Step 2), just 6 % translated goals into specific, measurable objectives. Best TA practices (Step 3) included adult learning tasks (5 %) and diffusion of innovation-oriented tasks (6 %). As part of the assessment of fit (Step 4), 27 % of articles assessed fit of TA with the recipient’s operations and organizational activities; yet, only one article assessed fit of TA with other support activities. As many as 78 % of articles assessed human capacity (TA providers’ expertise, knowledge, and skill) (Step 5), but just 2 % assessed technical capacity (e.g., video conferencing capabilities). Only 1 % of articles specified the development of a timeline for TA activities (Step 6). The most commonly reported metrics assessed as part of process evaluation (Step 7) were dosage (23 %), reach (25 %), and satisfaction (25 %), with much less assessing quality (6 %). Also, relatively few articles specified making mid-course corrections based on process evaluation findings (5 %). Thirty percent of articles described an outcome evaluation (Step 8) data collection process, and 78 % of articles reported the results of outcome evaluation. Outcome evaluation goals focused on general capacity (47 %) and/or innovation-specific capacity-building (41 %). Five percent used a plan-do-study-act process as part of engaging in continuous quality improvement (Step 9). To address sustainability (Step 10), 12 % integrated TA activities into the recipient’s delivery system.

## TA Relationships

As reported in Table 2, fifty-two (52/111 = 46.8 %) articles addressed TA relationships. More than a quarter of the articles (28/111 = 25.2 %) addressed the need for collaboration between TA providers and recipients. For example, one article mentioned a need for TA providers and recipients to develop a partnership and to have shared responsibility throughout the TA process (Corcoran and Robinson 1994). Fifteen articles (15/111 = 13.5 %) addressed being strengths-based, either to inspire the TA recipient or to provide positive reinforcement in connection with the recipient’s activities/behaviors. About 7 % (8/111) of the articles emphasized the necessity of having a trusting relationship. An article focused on the need for recipients to have trust that the TA providers will be there to help in a time of need (Cheadle et al. 2002).

## Innovation Life Cycle

Each article was coded according to one of three mutually exclusive life cycle stages: initiation (TA that is offered prior to implementing an innovation, with a focus on general capacity-building—e.g., leadership, supportive organizational climate), implementation (TA that is provided during the implementation of an innovation, with a focus on innovation-specific capacity-building—e.g., competencies for implementing an evidence-based intervention), and stability (TA that is provided subsequent to implementation of a specific innovation with an eye toward sustainability). As shown in Table 2, articles were nearly split in terms of being coded for initiation (47/111 = 42.3 %) versus implementation of an innovation (55/111 = 49.5 %), but few were coded as being in the stability stage (7/111 = 6.3 %). As a result of the small number of articles in the stability stage, articles in this particular stage were excluded from subsequent chi square analyses.^2^

Chi square and Fisher’s exact tests of independence were used to compare articles in the initiation stage with those in the implementation stage with respect to the reporting of tasks and relationships. Results of the comparison of the stages based on tasks are presented in Table 3. With the exception of one comparison, there were no significant differences between stages in the use of tasks. The only difference that was not based on chance variation was for Step 4 (assessment of fit) tasks. The proportions were significantly different, *X*^2^ (1, *N* = 38) = 5.40, *p* = 0.02, such that the assessment of TA fit was more likely to occur for supporting innovations in the initiation stage.

**Table 3:** TA tasks and relationships to support innovations in the initiation and implementation life cycle stages

Frames	Stage			
	Initiation	Implementation	Test statistic	*p*
	(*N* = 47) (%)	(*N* = 55) (%)		
Tasks				
TA needs and resources (Step 1)	32 (68.1)	36 (65.5)	*X* ^2^ = 0.08	0.78
TA goals and desired outcomes (Step 2)	41 (87.2)	49 (89.1)	*X* ^2^ = 0.08	0.77
Best TA practices (Step 3)	19 (40.4)	19 (34.5)	*X* ^2^ = 0.38	0.54
Fit (Step 4)^a^	15 (78.9)	8 (42.1)	*X* ^2^ = 5.40	0.02
Capacity (Step 5)	16 (84.2)	15 (78.9)	*X* ^2^ = 0.18	0.68
TA planning (Step 6)	3 (6.4)	7 (12.7)	*X* ^2^ = 1.15	0.28
Process evaluation (Step 7)	22 (46.8)	29 (52.7)	*X* ^2^ = 0.36	0.55
Outcome evaluation (Step 8)	39 (83.0)	42 (76.4)	*X* ^2^ = 0.68	0.41
Continuous quality improvement (Step 9)	7 (14.9)	6 (10.9)	*X* ^2^ = 0.36	0.55
Sustainability (Step 10)	11 (23.4)	18 (32.7)	*X* ^2^ = 1.08	0.30
Relationship feature				
Relationships addressed	24 (51.1)	24 (43.6)	*X* ^2^ = 0.56	0.45
Trust	6 (12.8)	2 (3.6)	Fisher’s	0.14
Respect	4 (8.5)	0 (0.0)	Fisher’s	0.04
Collaboration	17 (36.2)	10 (18.2)	*X* ^2^ = 4.21	0.04
Adjusting to readiness	0 (0.0)	1 (1.8)	–	–
Strengths-based	4 (8.5)	10 (18.2)	Fisher’s	0.25
Autonomy-supportive	4 (8.5)	4 (7.3)	Fisher’s	1.0
Roles	0 (0.0)	0 (0.0)	–	–
Rapport	2 (4.3)	2 (3.6)	Fisher’s	1.0

^a^The number of articles addressing best TA practices was used as the denominator for Steps 4 and 5

Results of the comparison of life cycle stages with respect to whether relationships were addressed are presented in Table 3. There were no differences between articles in initiation and implementation stages in whether relationships were addressed at all, *X*^2^ (1, *N* = 102) = 0.56, *p* = 0.45. However, there were some differences with respect to particular features of relationships. Articles in the initiation stage were significantly more likely to address collaboration than articles in the implementation stage, *X*^2^ (1, *N* = 102) = 4.21, *p* = 0.04. In addition, respect was significantly more likely to be addressed with articles about the initiation stage, Fisher’s exact test, *p* = 0.04. Being strengths-based was equally likely to be addressed in the initiation and implementation stages, Fisher’s exact test, *p* = 0.25, as was trust, Fisher’s exact test, *p* = 0.14.

## Discussion

The review of articles found very few instances in which an explicit model or organizing framework was used to plan, implement, and/or evaluate TA. Thus, although TA is widely practiced, it is not well defined or clearly operationalized into a series or sequence of tasks. It appears that the world of TA is like the early days of psychotherapy; there are inconsistencies in the literature, no agreement on the necessary ingredients, and relatively little use of systematic empirical evidence.

It is not surprising that when retrofitting GTO as a frame, high variability was observed in the extent to which particular tasks were used. For example, 87 % of articles generally addressed the goals and desired outcomes task (Step 2), but only seven articles operationalized goals into specific, measurable objectives. Therefore, it is hard to know what successful TA is, in a way that could be measured. Very few articles (9 %) specified TA planning (Step 6), which suggests that TA is largely reactive rather than being proactive and strategic. Process evaluation (Step 7) and outcome evaluation (Step 8) were used frequently, with variable rigor, whereas continuous quality improvement and sustainability were rarely utilized.

Notwithstanding such high variability, there are some strengths that can be built upon. As alluded to earlier, TA recipients do not only need capacity for implementing innovations; they also require motivation. A small set of articles specified best TA practices linked to diffusion of innovation theory (Rogers 2003) in which TA providers integrated motivational factors (e.g., trialability, relative advantage, simplicity) into communications with recipients. For instance, TA providers described the innovation as not being overly difficult to use (simplicity). In addition, articles specified the use of best TA practices connected to adult learning theory. For example, TA providers integrated frequent opportunities for interaction and engagement and made explicit efforts to link TA to the recipients’ own work and experiences.

Given that relationships are an essential part of TA (Butterfoss 2004; Wandersman et al. 2012), it is not surprising that relationships were addressed in about half of the reviewed articles. Collaboration, strengths-based, and trust were the most frequently mentioned relationship features. The fact that collaboration was frequently specified in articles as a relationship feature suggests that when attention is given to relationships, there is likely to be an effort to avoid setting up a situation where recipients are just passive. Instead of being “empty vessels” to be filled, TA recipients are active agents who are always learning and growing and could themselves serve as a resource to others in the future.

Having a trusting TA relationship was also emphasized in many articles. When TA providers have a quality assurance role, recipients may have the perception that it is risky to be fully candid (Mitchell et al. 2002), which, in turn, can limit the extent to which TA can be helpful. Therefore, establishing trust (which could include full disclosure about the limits of confidentiality) has value in terms of helping to temper some reluctance that recipients may have about sharing sensitive information about areas requiring support (Chen 2001; O’Sullivan and O’Sullivan 1998).

Being strengths-based appears to be another essential part of the TA relationship, as recipients often have doubts and anxiety when embarking on something new. A relationship that is strengths-based can help to build the recipient’s self-efficacy (Bandura 1994) or confidence in successively executing an innovative practice. Although the strongest source of confidence is actually mastering the behavior, until this happens, it is helpful for TA providers to build recipients’ confidence through a strengths-based relationship.

In relation to the life cycle frame, only a small number of articles described providing TA for recipients at the stability stage of an innovation, suggesting that TA providers did not tend to work with recipients who already had an innovation long in place and were now seeking to sustain it. Providing TA at the stability stage is important to ensure lasting gains in capacity (Simmons et al. 2011; Stillman et al. 2013). An important role for TA providers would involve helping to ensure that capacity built through TA is sustainable. The fact that there were so few differences by life cycle stage is reflective of the high variability reported earlier.

Variance in the use of TA tasks is mostly independent of where the recipients are in terms of the innovation’s life cycle. One exception involves the assessment of fit (GTO Step 4); this step was significantly more likely to be reported for articles in the initiation stage. This association speaks to the fact that issues of fit between TA services and the recipient are especially important when working at a more foundational stage (Cherniss 1993; Thomas et al. 1997).

There were some differences between stages in terms of the reporting of relationship features. The most robust finding was a difference in collaboration; this feature was emphasized more in articles describing the initiation stage of the life cycle. The decreased emphasis on collaboration in the implementation stage does not necessarily mean that the TA recipients have less of a voice. It may be that there is less collaboration because TA providers may let go and allow the recipients to have more control over the direction of the TA process (e.g., decisions about which knowledge and skills to work on) as the recipients develop greater capacity (Fawcett et al. 1995). Having a respectful relationship was also significantly more likely to be emphasized at the initiation stage. Although having a respectful relationship would seemingly be important at all stages, it appears that respect is so foundational that if this feature were to be absent, subsequent stages in TA would either not occur or would occur but not be successful (Tang et al. 2005).

## Limitations

One potential limitation of this synthesis relates to whether the items being coded were implicitly versus explicitly mentioned in articles. The fact that an article does not mention a task (GTO step) or relationship feature does not mean that it was not actually addressed in the work. For example, it may be that a TA project had a goal guiding the work but this goal was not explicitly stated in the article. In this case, the article would not be coded for goals (GTO Step 2), although there may have been (unreported) goal setting. Another limitation is that the search strategy may have missed other relevant articles that describe the provision of TA to support innovations. For example, including the term “professional development” in the search strategy might have yielded other articles about TA. In addition, a finite number of search engines were utilized; the inclusion of other search engines (e.g., ERIC) may have allowed for identification of other articles that meet the inclusion criteria for the synthesis.

A third limitation is that the innovation life cycle model used as a frame for this synthesis assumes that it is normal for TA providers to work with recipients at an initiation stage, followed by working with them at an implementation stage and finally at a stability stage. But only a small handful of articles reported that TA providers worked with recipients at more than one of these stages. As a next step, it would be useful to identify a small set of articles reporting more than one stage and conduct a multiple case study (Stake 2013) in order to more deeply assess TA according to the innovation life cycle.

## Implications for Future Research and Practice

Additional models/organizing approaches are needed so that TA research and practice can develop more systematically. The use of GTO as a frame in this synthesis is an example of a model/organizing approach. To further translate GTO-TA into an empirically supported practice, it will be important to develop a structured GTO-TA intervention that can be empirically tested in relation to a control or comparison group to determine if specific outcomes have improved as a result of GTO-TA. Field research can also be conducted on each of the steps to identify best practices which can be disseminated to other TA providers working in the field.

There are additional implications for conducting research with the data collected for this synthesis. For example, it would be useful to do some additional stratification of the articles to permit deeper information about similarities and differences in TA across different kinds of situations in which TA is typically delivered. This includes both the level of analysis (e.g., community versus organization) for TA delivery as well as the content area for the innovation (e.g., HIV/AIDS, youth development, etc.) that is being supported.

In terms of implications for practice, there is currently a great deal of ambiguity about what constitutes high-quality TA. TA providers tend to rely on word-of-mouth and anecdotal experience, in contrast to referring to a widely agreed upon set of standards. As discussed in the next section, a need exists for standards to enhance quality and accountability in TA.

## On the Need for Standards to Enhance Quality and Accountability in TA

The findings from the synthesis—particularly under the tasks frame—are worrisome. Although much money and time is invested in TA, TA is often delivered with insufficient rigor. What can be done to increase the extent to which TA providers are embodying exemplary practices within each of the three frames in this synthesis (tasks, relationships, and life cycle)?

There is a noticeable absence of widely recognized standards for high-quality TA. Having such standards available would allow for an objective perspective about quality to guide decision-making about necessary improvements and provide a lens for making judgments about whether TA was properly executed. The standards should include items relevant to the three frames in this synthesis: strategically using the right tasks, having quality relationships, and appropriately adjusting TA to the life cycle stage of the innovation being supported. In addition to making such standards widely available, there should be oversight to ensure that the standards are properly brought into practice. Agencies or foundations that are funding TA contractors should design requests for proposals around these standards, and an important part of the evaluation of the proposal should focus on the extent to which the standards are reflected in the proposed plan for TA delivery. In addition, the evaluation of the funded contractor’s TA delivery should focus on the extent to which TA standards are accomplished.

While standards usually exist for the delivery system’s implementation of innovations, it is rare for there to be standards for the support system’s implementation of TA. Having TA standards available and reinforced should help remediate many of the gaps noted in this synthesis.

## Conclusion

The findings of this synthesis indicate that TA needs to be provided much more systematically. In order to move the field of TA in this direction and to ensure greater quality and accountability, it is necessary to develop standards for high-quality TA. We see strong parallels between this need and the evolution of psychotherapy in the direction of evidence-supported therapies. The frames used in this synthesis (tasks, relationships, and innovation life cycle) can be used as a starting point for identifying standards for which TA providers should be held accountable. Funders can integrate these standards into TA requirements and should ensure that the standards are appropriately embodied in TA practice.

## Electronic Supplementary Material

Below is the link to the electronic supplementary material.

ESM 1 (DOCX 242 kb)

ESM 1 (DOCX 75 kb)

ESM 1 (DOCX 64 kb)

ESM 1 (DOCX 28 kb)

ESM 1 (DOCX 13 kb)

## Footnotes

1 Getting To Outcomes and GTO are trademarks registered by the University of South Carolina and RAND.

2 Two articles did not address the life cycle, and 16 articles were coded according to more than one of these stages because of the fact that the author traced the evolution of the innovation retrospectively. More specifically, ten addressed both initiation and implementation; five addressed both implementation and stability; and one addressed both initiation and stability. As a rule of thumb, the more recent stage was preserved in cases of duplication (e.g., implementation was used when initiation and implementation were both reported). The de-duplication was done in order to ensure that the assumption of independent observations for the independent samples chi square test was not violated. A sensitivity analysis was performed to identify the extent to which the chi square results would change when the least recent phase was used, and no differences were observed. The de-duplicated frequencies for the life cycle stages are presented in Table 2.
