# Supplementary material for: Technical Assistance to Enhance Prevention Capacity: a Research Synthesis of the Evidence Base
Source: Prev Sci. 2016 Feb 9;17:417–28. doi: 10.1007/s11121-016-0636-5 (PMC4839040; doi:10.1007/s11121-016-0636-5)
Supplement: Supplementary file 1 — (DOCX 242 kb) [file 11121_2016_636_MOESM1_ESM.docx]

**Supplementary Material: List of Reviewed Articles**

|  | Ackermann, R.T. (2010). Description of an integrated framework for building linkages among primary care clinics and community organizations for the prevention of type 2 diabetes: emerging themes from the CC-Link study. *Chronic Illness, 6*(89), 89-100. |
| --- | --- |
|  | Adsit, R., Fraser, D., Redmond, L., Smith, S., & Fiore, M. (2005). Changing clinical practice, helping people quit: the Wisconsin Cessation Outreach Model. *Wisconsin Medical Journal, 104*(4), 32-26. |
|  | Andrews, A. P., Vincent, M. L., Fawcett, S. B., Campuzano, M. K., Harris, K. J., Lewis, R. K., Williams, E. L., & Fisher, J. L. (1996). Replicating a community initiative for preventing adolescent pregnancy: from South Carolina to Kansas. *Family & Community Health, 19*(1), 14-30. |
|  | August, G.J., Bloomquist, M.L., Lee, S.S., Realmuto, G.M., & Hektner, J.M. (2006). Can evidence-based prevention programs be sustained in community practice settings? The Early Risers’ Advanced-Stage Effectiveness Trial. *Prevention Science, 7*(2), 151-165. |
|  | Ayala, G., Chion, M., Diaz, R.M., Heckert, A.L., Nuno, M., del Pino, H.E., Rodriguez, C., Schroeder, K., & Smith, T. (2007). Accion Mutua (Shared Action): A multipronged approach to delivering capacity-building assistance to agencies serving Latino communities in the United States. *Journal of Public Health Management Practice, January*(Suppl), S33-S39. |
|  | Beam, M., Ehrlich, G., Donze Black, J., Block, A., & Leviton, L. (2012). Evaluation of the Healthy Schools Program: Part II. The role of technical assistance. *Preventing Chronic Disease, 9*, 1-6. |
|  | Bernstein, S.L., Jearld, S., Prasad, D., Bax, P., & Bauer, U. (2009). Rapid implementation of a smokers’ quitline fax referral service in an urban area. *Journal of Health Care for the Poor and Underserved, 20*, 55-63. |
|  | Braun, K.L., Tsark, J., Santos, L.A., & Abrigo, L. (2003a). ‘Imi Hale – The Native Hawaiian Cancer Awareness, Research, and Training Network: second-year status report. *Asian American Pacific Islander Journal of Health, 10*(1), 4–16. |
|  | Braun, K. L., Ichiho, H. M., Kuhaulua, R. L., Aitaoto, N. T., Tsark, J. U., Spegal, R., & Lamb, B. M. (2003b). Empowerment through community building: Diabetes Today in the Pacific. *Journal of Public Health Management and Practice, 9,* S19-S25. |
|  | Brock, J., Mitchell, J., Irby, K., Stevens, B., Archibald, T., Goroski, A., & Lynn, J. (2013). Association between quality improvement for care transitions in communities and rehospitalizations among Medicare beneficiaries. *Jama, 309(4)*, 381-391. |
|  | Brown, L. F., Keily, P. A., & Spencer, A. J. (1994). Evaluation of a continuing education intervention “Periodontics in general practice”. *Community Dentistry and Oral Epidemiology, 22*(6), 441-447. |
|  | Brown, D. R., Scott, W., Lacey, K., Blount, J., Roman, D., & Brown, D. (2006). Black Churches in Substance Use and Abuse Prevention Efforts. *Journal of Alcohol and Drug Education, 50*(2), 43-65. |
|  | Brownson, R. C., Ballew, P., Brown, K. L., Elliott, M. B., Haire-Joshu, D., Heath, G. W., & Kreuter, M. W. (2007). The effect of disseminating evidence-based interventions that promote physical activity to health departments. *American Journal of Public Health, 97*(10), 1900. |
|  | Buller, D.B., Reynolds, J.L., Ashley, M.K., Buller, I.L., Kane, C.L., Stabell, K.L., Massie, X.L., & Cutter, G.R. (2011). Motivating public school districts to adopt sun protection policies: A randomized controlled trial. *American Journal of Preventive Medicine, 41*(3), 309-316. |
|  | Butcher, M.K., Gilman, J., Meszaros, J.F., Bjorsness, D., Madison, M., McDowall, J.M., Oser, C.S., Johnson, E.A., Harwell, T.S., Helgerson, S.D., & Gohdes, D. (2006). Improving Access to Quality Diabetes Education in a Rural State The Montana Quality Diabetes Education Initiative. *The Diabetes Educator, 32*(6), 963-967. |
|  | Butterfoss, F. D. (2004). The coalition technical assistance and training framework: helping community coalitions help themselves. *Health Promotion Practice, 5*(2), 118-126. |
|  | Carlson, J.S., Mackrain, M.A., Van Egeren, L.A., Brophy-Herb, H., Kirk, R.H., Marciniak, D., Falvay, S., Zheng, Y., Bender, S.L., Tableman, B. (2012). Implementing a statewide early childhood mental health consultation approach to preventing childcare expulsion. *Infant Mental Health Journal 33*(3), 265-273. |
|  | Chamberlain, L. (2008). Ten lessons learned in Alaska: Home visitation and intimate partner violence. *Journal of emotional abuse, 8*(1-2), 205-216. |
|  | Cheadle, A., Sullivan, M., Krieger, J., Ciske, S., Shaw, M., Schier, J. K., & Eisinger, A. (2002). Using a participatory approach to provide assistance to community-based organizations: the Seattle Partners Community Research Center. *Health Education & Behavior, 29*(3), 383-394. |
|  | Chinman, M., Hunter, S. B., Ebener, P., Paddock, S. M., Stillman, L., Imm, P., & Wandersman, A. (2008). The getting to outcomes demonstration and evaluation: An illustration of the prevention support system. *American Journal of Community Psychology, 41*(3-4), 206-224. |
|  | Chinman, M., Hannah, G., & McCarthy, S. (2012). Lessons learned from a quality improvement intervention with homeless veteran services. *Journal of Health Care for the Poor and Underserved, 23*(3), 210-224. |
|  | Chinman, M., Acosta, J., Ebener, P., Burkhart, Q., Malone, P.S., Paddock, S.M., Clifford, M., Corsello, M., Duffey, T., Hunter, S., Jones, M., Lahti, M., Phillips, A., Savell, S., Scales, P.C., & Tellett-Royce, N. (2013). Intervening with practitioners to improve the quality of prevention: one-year findings from a randomized trial of Assets-Getting To Outcomes. *The Journal of Primary Prevention, 34*(3), 173-191. |
|  | Collins, C., Harshbarger, C., Sawyer, R., & Hamdallah, M. (2006). The diffusion of effective behavioral interventions project: development, implementation, and lessons learned. *AIDS Education & Prevention, 18*(supp), 5-20. |
|  | Corcoran, R. D., & Robinson, R. G. (1994). Community demonstration project initiative: technical support for program development at the community level. *Cancer Practice, 2*(3), 222-228. |
|  | Cousins, J. M., Langer, S. M., Thomas, C., & Rhew, L. K. (2011). The role of state health departments in supporting community-based obesity prevention. *Preventing Chronic Disease, 8*(4), 1-10. |
|  | Cura, E. N., & Segura, E. L. (1998). Quality assurance of the serologic diagnosis of Chagas' disease. *Pan American Journal of Public Health, 3*(4), 242-248. |
|  | Curtis, L., Brown, Z. G., & Gill, J. E. (2008). Sisters Together: Move More, Eat Better: a community-based health awareness program for African-American women. *Journal of National Black Nurses' Association, 19*(2), 59-64. |
|  | De Rosa, C.J., Jeffries, R.A., Afifi, A.A., Cumberland, W.G., Chung, E.Q., Kerndt, P.R., Ethier, K.A., Martinez, E., Loya, R.V., & Dittus, P.J. (2012). Improving the implementation of a condom availability program in urban high schools. *Journal of Adolescent Health, 51*(6), 572-579. |
|  | Dick, R. W., Manson, S. M., Hansen, A. L., Huggins, A., & Trullinger, L. (2007). The Native Telehealth Outreach and Technical Assistance Program: a community-based approach to the development of multimedia-focused health care information. *American Indian and Alaska Native Mental Health Research: The Journal of the National Center, 14*(2), 49-66. |
|  | Ealey, S., Wituk, S., Schultz, J., Usher, J., Meissen, G., & Pearson, R. (2006). The Community Development for Healthy Children Initiative. *Journal of Community Practice, 14*(4), 129-138. |
|  | Evans, A. Jr., Okeke, B., Ali, S., Achara-Abrahams, I., OHara, T., Stevenson, T., Warner, N., Bolton, C., Lim, S., Faith, J., King, J., Davidson, L., Poplawski, P., Rothbard, A., & Salzer, M. (2012). Converting partial hospitals to community integrated recovery centers. *Community Mental Health Journal, 48*(5), 557-563. |
|  | Fagan, A. A., Hanson, K., Briney, J. S., & Hawkins, J. D. (2012). Sustaining the utilization and high quality implementation of tested and effective prevention programs using the Communities That Care prevention system. *American Journal of Community Psychology, 49*(3-4), 365-377. |
|  | Fernald, D., Harris, A., Deaton, E. A., Weister, V., Pray, S., Baumann, C., & Levinson, A. (2012). A standardized reporting system for assessment of diverse public health programs*. Preventing Chronic Disease, 9*, n.p. |
|  | Florin, P., Celebucki, C., Stevenson, J., Mena, J., Salago, D., White, A., Harvey, B., & Dougal, M. (2006). Cultivating systemic capacity: the Rhode Island tobacco control enhancement project. *American Journal of Community Psychology, 38*(3-4), 213-220. |
|  | Fouad, M.N., Partridge, E., Dignan, M., Holt, C., Johnson, R., Nagy, C., Parha, G., Person, S., Scarinci, I., & Wynn, T. (2005). A community-driven action plan to eliminate breast and cervical cancer disparity: successes and limitations. Journal of Cancer Education, 21(1 Suppl), S91-100. |
|  | Fourney, A., Gregson, J., Sugerman, S., & Bellow, A. (2011). Building evaluation capacity in local programs for multisite nutrition education interventions. *Journal of Nutrition Education and Behavior, 43*(4), S130-S136. |
|  | Friedsam, D., Haug, G., Rust, M., & Lake, A. (2003). Tribal benefits counseling program: expanding health care opportunities for tribal members. *American Journal of Public Health, 93*(10), 1634-1636. |
|  | Geiger, B. F., Petri, C. J., Myers, O., Lan, J., Binkley, D., Aldige, C. R., & Berdebes, J. (2002). Using technology to teach health: a collaborative pilot project in Alabama. *Journal of School Health, 72*(10), 401-407. |
|  | Gibbs, D. A., Hawkins, S. R., Clinton-Sherrod, A. M., & Noonan, R. K. (2009). Empowering programs with evaluation technical assistance outcomes and lessons learned. *Health Promotion Practice, 10*(1 suppl), 38S-44S. |
|  | Gilliam, A., Barrington, T., Davis, D., Lacson, R., Uhl, G., & Phoenix, U. (2003). Building evaluation capacity for HIV prevention programs. *Evaluation and Program Planning, 26*(2), 133-142. |
|  | Glang, A., Tyler, J., Pearson, S., Todis, B., & Morvant, M. (2004). Improving educational services for students with TBI through statewide consulting teams. *NeuroRehabilitation, 19*(3), 219-231. |
|  | Grisham-Brown, J., Hallam, R. A., & Pretti-Frontczak, K. (2008). Preparing Head Start personnel to use a curriculum-based assessment: an innovative practice in the “age of accountability.” *Journal of Early Intervention, 30*(4), 271-281. |
|  | Hannon, P.A., Harris, J.R., Sopher, C.J., Kuniyuki, A., Ghosh, D.L., Henderson, S., Martin, D.P., Weaver, M.R., Williams, B., Albano, D.L., Hammerback, K.E., Parks, M.R., Forehand, M., Meischke, H., Diehr, P., & Lichiello, P. (2012). Improving low-wage, midsized employers' health promotion practices: a randomized controlled trial. *American Journal of Preventive Medicine, 43*(2), 125-133. |
|  | Hanson, L. C., Reynolds, K. S., Henderson, M., & Pickard, C. G. (2005). A quality improvement intervention to increase palliative care in nursing homes. *Journal of Palliative Medicine, 8*(3), 576-584. |
|  | Hawkins, J. D., Oesterle, S., Brown, E. C., Arthur, M. W., Abbott, R. D., Fagan, A. A., & Catalano, R. F. (2009). Results of a type 2 translational research trial to prevent adolescent drug use and delinquency: a test of Communities That Care. *Archives of Pediatrics & Adolescent Medicine, 163*(9), 789-798. |
|  | Hawkins, J. D., Oesterle, S., Brown, E. C., Monahan, K. C., Abbott, R. D., Arthur, M. W., & Catalano, R. F. (2012). Sustained decreases in risk exposure and youth problem behaviors after installation of the Communities That Care prevention system in a randomized trial. *Archives of Pediatrics & Adolescent Medicine, 166*(2), 141-148. |
|  | Hessel, A. S., Marshall, J. W., Sabina, A. B., & DeForest, K. (2010). Healthy & Active Communities Initiative: A foundation's response to the obesity epidemic. *Preventive Medicine, 50,* S93-S94. |
|  | Honeycutt, S., Carvalho, M., Glanz, K., Daniel, S. D., & Kegler, M. C. (2012). Research to reality: a process evaluation of a mini-grants program to disseminate evidence-based nutrition programs to rural churches and worksites. *Journal of Public Health Management and Practice, 18*(5), 431-439. |
|  | Horne, L., Miller, K., Silva, S., & Anderson, L. (2013). Implementing the ACHIEVE Model to Prevent and Reduce Chronic Disease in Rural Klickitat County, Washington. *Preventing Chronic Disease, 10*, 1-9. |
|  | Horner, J. K., Hanson, L. C., Wood, D., Silver, A. G., & Reynolds, K. S. (2005). Using quality improvement to address pain management practices in nursing homes. *Journal of Pain and Symptom Management, 30*(3), 271-277. |
|  | Horner, R. H., Sugai, G., Smolkowski, K., Eber, L., Nakasato, J., Todd, A. W., & Esperanza, J. (2009). A randomized, wait-list controlled effectiveness trial assessing school-wide positive behavior support in elementary schools. *Journal of Positive Behavior Interventions*, *11*(3), 133-144. |
|  | Hunter, S. B., Chinman, M., Ebener, P., Imm, P., Wandersman, A., & Ryan, G. W. (2009). Technical assistance as a prevention capacity-building tool: a demonstration using the Getting To Outcomes^TM^ Framework. *Health Education & Behavior, 36*(5), 810-828. |
|  | Jackson, C., Fortmann, S. P., Flora, J. A., Melton, R. J., Snider, J. P., & Littlefield, D. (1994). The capacity-building approach to intervention maintenance implemented by the Stanford Five-City Project. *Health Education Research, 9*(3), 385-396. |
|  | Johnson, K., Collins, D., & Wandersman, A. (2013). Sustaining innovations in community prevention systems: a data-informed sustainability strategy. *Journal of Community Psychology, 41*(3), 322-340. |
|  | Kahn, L., Hurth, J., Kasprzak, C. M., Diefendorf, M. J., Goode, S. E., & Ringwalt, S. S. (2009). The National Early Childhood Technical Assistance Center model for long-term systems change. *Topics in Early Childhood Special Education, 29*(1), 24-39. |
|  | Kamara, A. (1997). Developing a network: the PMM process. *International Journal of Gynecology & Obstetrics, 59*, S27-S35. |
|  | Kegler, M. C., & Redmon, P. B. (2006). Using technical assistance to strengthen tobacco control capacity: evaluation findings from the tobacco technical assistance consortium. *Public Health Reports, 121*(5), 547-556. |
|  | Kelly, J.A., Somlai, A.M., DiFranceisco, W.J., Otto-Salaj, L.L., McAuliffe, T.L., Hackl, K.L., Heckman, T.G., Holtgrave, D.R., & Rompa, D. (2000). Bridging the gap between the science and service of HIV prevention: transferring effective research-based HIV prevention interventions to community AIDS service providers. *American Journal of Public Health 90*(7), 1082-1088. |
|  | Kennedy, C., Finkelstein, N., Hutchins, E., & Mahoney, J. (2004). Improving screening for alcohol use during pregnancy: The Massachusetts ASAP program. *Maternal and Child Health Journal, 8*(3), 137-147. |
|  | Klein, J., & Nelson, D. (2000). Homeownership for people with disabilities: The state of the States in 1999. *Journal of Vocational Rehabilitation, 15*(2), 67-77. |
|  | Kreger, M., Sargent, K., Arons, A., Standish, M., & Brindis, C. D. (2011). Creating an environmental justice framework for policy change in childhood asthma: a grassroots to treetops approach. *Journal Information, 101*(S1), S208-216. |
|  | Kumpfer, K. L., Pinyuchon, M., de Melo, A. T., & Whiteside, H. O. (2008). Cultural adaptation process for international dissemination of the Strengthening Families Program. *Evaluation & the Health Professions, 31*(2), 226-239. |
|  | Lazovich, D., Parker, D. L., Brosseau, L. M., Milton, F. T., Dugan, S. K., Pan, W., & Hock, L. (2002). Effectiveness of a worksite intervention to reduce an occupational exposure: the Minnesota wood dust study. *American Journal of Public Health, 92*(9), 1498-1505. |
|  | Leake, R., Green, S., Marquez, C., Vanderburg, J., Guillaume, S., & Gardner, V. A. (2007). Evaluating the capacity of faith-based programs in Colorado. *Research on Social Work Practice, 17*(2), 216-228. |
|  | Lee, J. G., Ranney, L. M., Goldstein, A. O., McCullough, A., Fulton-Smith, S. M., & Collins, N. O. (2011). Successful implementation of a wellness and tobacco cessation curriculum in psychosocial rehabilitation clubhouses*. BMC Public Health, 11*(1), 702-712. |
|  | Leviton, L. C., Herrera, C., Pepper, S. K., Fishman, N., & Racine, D. P. (2006). Faith in action: capacity and sustainability of volunteer organizations. *Evaluation and Program Planning, 29*(2), 201-207. |
|  | Lim Brodowski, M., Counts, J. M., Gillam, R. J., Baker, L., Spiva Collins, V., Winkle, E., Skala, J., Stokes, K., Gomez, R., & Redmon, J. (2013). Translating evidence-based policy to practice: A multilevel partnership using the interactive systems framework. *Families in Society: The Journal of Contemporary Social Services, 94*(3), 141-149. |
|  | Luiselli, J. K., Putnam, R. F., Handler, M. W., & Feinberg, A. B. (2005). Whole‐school positive behaviour support: effects on student discipline problems and academic performance. *Educational Psychology, 25*(2-3), 183-198. |
|  | Lyons, C. W. (1986). Interagency alliances link young and old. *Children Today, 15*(5), 21-25. |
|  | Mann, T. L., Boss, J., & Randolph, S. (2007). Pathways to prevention: a training and technical assistance initiative to increase program capacity to address infant mental health issues in Early Head Start. *Infant Mental Health Journal, 28*(2), 106-129. |
|  | Materna, B. L., Harrington, B., Scholz, P., Payne, S. F., Stubbs, H. A., Hipkins, K., Merideth, E., Kirsch, L., Lomax, G., Coyle, P., & Uratsu, C. (2002). Results of an intervention to improve lead safety among painting contractors and their employees. *American Journal of Industrial Medicine, 41*(2), 119-130. |
|  | Mayberry, R. M., Daniels, P., Yancey, E. M., Akintobi, T. H., Berry, J., Clark, N., & Dawaghreh, A. (2009). Enhancing community-based organizations’ capacity for HIV/AIDS education and prevention. *Evaluation and Program Planning, 32*(3), 213-220. |
|  | McClellan, W. M., Hodgin, E., Pastan, S., McAdams, L., & Soucie, M. (2004). A randomized evaluation of two health care quality improvement program (HCQIP) interventions to improve the adequacy of hemodialysis care of ESRD patients: feedback alone versus intensive intervention. *Journal of the American Society of Nephrology, 15*(3), 754-760. |
|  | Mercer, M. A., Gates, N., Holley, M., Malunga, L., & Arnold, R. (1996). Rapid KABP survey for evaluation of NGO HIV/AIDS prevention projects. *AIDS Education and Prevention, 8*(2), 143-154. |
|  | Miller, K. S., Maxwell, K. D., Fasula, A. M., Parker, J. T., Zackery, S., & Wyckoff, S. C. (2010). Pre-risk HIV-prevention paradigm shift: the feasibility and acceptability of the Parents Matter! Program in HIV risk communities. *Public Health Reports, 125*(Suppl 1), 38-46. |
|  | Mitchell, R. E., Stone-Wiggins, B., Stevenson, J. F., & Florin, P. (2004). Cultivating capacity: outcomes of a statewide support system for prevention coalitions. *Journal of Prevention & Intervention in the Community, 27*(2), 67-87. |
|  | Naylor, M. W., Anderson, T. R., & Morris, A. (2003). Child psychiatry and child welfare: A collaboration for change. *Residential Treatment for Children & Youth, 21*(1), 33-50. |
|  | Needle, R. H., Trotter, R. T., Singer, M., Bates, C., Page, J. B., Metzger, D., & Marcelin, L. H. (2003). Rapid assessment of the HIV/AIDS crisis in racial and ethnic minority communities: an approach for timely community interventions. *American Journal of Public Health, 93*(6), 970-979. |
|  | Nemec, P. B., Forbess, R., Cohen, M. R., Farkas, M. D., Rogers, E. S., & Anthony, W. (1991). Effectiveness of technical assistance in the development of psychiatric rehabilitation programs. *The Journal of Mental Health Administration, 18*(1), 1-11. |
|  | Newton, J. S., Algozzine, B., Algozzine, K., Horner, R. H., & Todd, A. W. (2011). Building local capacity for training and coaching data-based problem solving with positive behavior intervention and support teams. *Journal of Applied School Psychology, 27*(3), 228-245. |
|  | Northup, J., Wacker, D. P., Berg, W. K., Kelly, L., Sasso, G., & DeRaad, A. (1994). The treatment of severe behavior problems in school settings using a technical assistance model. *Journal of Applied Behavior Analysis, 27*(1), 33-47. |
|  | Oliva, G., Rienks, J., & Chavez, G. F. (2007). Evaluating a program to build data capacity for core public health functions in local maternal child and adolescent health programs in California. *Maternal and Child Health Journal, 11*(1), 1-10. |
|  | Perka, E. J. (2011). Culture change in addictions treatment: a targeted training and technical assistance initiative affects tobacco-related attitudes and beliefs in addiction treatment settings. *Health Promotion Practice, 12*(6 suppl 2), 159S-165S. |
|  | Plescia, M., Young, S., & Ritzman, R. L. (2005). Statewide community-based health promotion: a North Carolina model to build local capacity for chronic disease prevention. *Preventing Chronic Disease, 2*, 1-8. |
|  | Polacsek, M., O’Brien, L. M., Lagasse, W., & Hammar, N. (2006). Move & Improve: a worksite wellness program in Maine. *Preventing Chronic Disease, 3*(3), 1-8. |
|  | Randolph, F., Blasinsky, M., Morrissey, J. P., Rosenheck, R. A., Cocozza, J., & Goldman, H. H. (2002). Overview of the ACCESS program. *Psychiatric Services, 53*(8), 945-948. |
|  | Rantz, M. J., Vogelsmeier, A., Manion, P., Minner, D., Markway, B., Conn, V., Aud, M. A., & Mehr, D. R. (2003). Statewide strategy to improve quality of care in nursing facilities. *The Gerontologist 43*(2), 248-258. |
|  | Redmond, L. A., Adsit, R., Kobinsky, K. H., Theobald, W., & Fiore, M. C. (2010). A decade of experience promoting the clinical treatment of tobacco dependence in Wisconsin. *Wisconsin Medical Journal, 109*(2), 71-78. |
|  | Rhew, I. C., Brown, E. C., Hawkins, J. D., & Briney, J. S. (2013). Sustained effects of the Communities That Care system on prevention service system transformation. *American Journal of Public Health, 103*(3), 529-535. |
|  | Roeseler, A., Hagaman, T., & Kurtz, C. (2011). The use of training and technical assistance to drive and improve performance of California’s Tobacco Control Program. *Health Promotion Practice, 12*(2), 130S–143S. |
|  | Rogers, E. S., Cohen, B. F., Danley, K. S., Hutchinson, D., & Anthony, W. A. (1986). Training mental health workers in psychiatric rehabilitation. *Schizophrenia Bulletin, 12*(4), 709-719. |
|  | Rohrbach, L. A., Gunning, M., Sun, P., & Sussman, S. (2010). The Project Towards No Drug Abuse (TND) Dissemination Trial: implementation fidelity and immediate outcomes. *Prevention Science, 11*(1), 77-88. |
|  | Rose, E. (1991). Project TAPE: a model of technical assistance for service providers of college students with learning disabilities. *Learning Disabilities Research & Practice, 6*, 25-33. |
|  | Sahyoun, N. R., Akobundu, U., Coray, K., & Netterville, L. (2009). Challenges in the delivery of nutrition services to hospital discharged older adults: the community connections demonstration project. *Journal of Nutrition for the Elderly, 28*(2), 127-142. |
|  | Satcher, D., Sullivan, L. W., Douglas, H. E., Mason, T., Phillips, R. F., Sheats, J. Q., & Smith, S. A. (2006). Enhancing cancer control programmatic and research opportunities for African‐Americans through technical assistance training. *Cancer, 107*(S8), 1955-1961. |
|  | Scholl, K. G., Smith, J. G., & Davison, A. (2005). Agency readiness to provide inclusive recreation and after-school services for children with disabilities. *Therapeutic Recreation Journal, 39*(1), 47-62. |
|  | Sheffer, M. A., Baker, T. B., Fraser, D. L., Adsit, R. T., McAfee, T. A., & Fiore, M. C. (2012). Fax referrals, academic detailing, and tobacco quitline use: a randomized trial. *American Journal of Preventive Medicine, 42*(1), 21-28. |
|  | Shoemaker, P. A., Skogstrom, T., Shea, J., & Bethune, L. (2007). The Boston Safe Shops Project--preliminary findings of a case study in applying the 10 essential services of public health to building environmental health capacity. *Journal of Environmental Health, 70*(1), 22. |
|  | Sigafoos, J., Kigner, J., Holt, K., Doss, S., & Mustonen, T. (1991). Improving the quality of written developmental policies for adults with intellectual disabilities. *The British Journal of Mental Subnormality, 37*(72), 35-46. |
|  | Spoth, R., Guyll, M., Redmond, C., Greenberg, M., & Feinberg, M. (2011). Six-year sustainability of evidence-based intervention implementation quality by community-university partnerships: the PROSPER study. *American Journal of Community Psychology, 48*(3-4), 412-425. |
|  | Striffler, N., Perry, D. F., & Kates, D. A. (1997). Planning and implementing a finance system for early intervention services. *Infants & Young Children, 10*(2), 57-65. |
|  | Sullivan, W. P., & Rapp, C. A. (1991). Improving client outcomes: The Kansas technical assistance consultation project. *Community Mental Health Journal, 27*(5), 327-336. |
|  | Tompkins, N. O. H., Rye, J. A., Zizzi, S., & Vitullo, E. (2005). Engaging rural youth in physical activity promotion research in an after-school setting. *Preventing Chronic Disease, 2*, 1-7. |
|  | Valente, T. W., Chou, C. P., & Pentz, M. A. (2007). Community coalitions as a system: effects of network change on adoption of evidence-based substance abuse prevention. *American Journal of Public Health, 97*(5), 880-886. |
|  | Ward, D. S., Benjamin, S. E., Ammerman, A. S., Ball, S. C., Neelon, B. H., & Bangdiwala, S. I. (2008). Nutrition and physical activity in child care: results from an environmental intervention. *American Journal of Preventive Medicine, 35*(4), 352-356. |
|  | Washington, A. E., Nápoles-Springer, A., Forté, D. A., Alexander, M., & Pérez-Stable, E. J. (2002). Establishing centers to address treatment effectiveness in diverse ethnic groups: the MEDTEP experience. *Ethnicity and Health, 7*(4), 231-242. |
|  | Watson-Thompson, J., Woods, N. K., Schober, D. J., & Schultz, J. A. (2013). Enhancing the capacity of substance abuse prevention coalitions through training and technical assistance. *Journal of Prevention & Intervention in the Community, 41*(3), 176-187. |
|  | Weiler, R. M., Pigg, R. M., & McDermott, R. J. (2003). Evaluation of the Florida coordinated school health program pilot schools project. *Journal of School Health, 73*(1), 3-8. |
|  | Wesley, P. W., & Buysse, V. (1996). Supporting early childhood inclusion lessons learned through a statewide technical assistance project. *Topics in Early Childhood Special Education, 16*(4), 476-499. |
|  | Wilcox, S., Laken, M., Parrott, A. W., Condrasky, M., Saunders, R., Addy, C. L., Evans, R., Baruth, M., & Samuel, M. (2010). The faith, activity, and nutrition (FAN) program: design of a participatory research intervention to increase physical activity and improve dietary habits in African American churches. *Contemporary Clinical Trials, 31*(4), 323-335. |
|  | Young, A. S., Chinman, M., Forquer, S. L., Knight, E. L., Vogel, H., Miller, A., Rowe, M., & Mintz, J. (2005). Use of a consumer-led intervention to improve provider competencies. *Psychiatric Services, 56*(8), 967-975. |
