# Supplementary material for: Technical Assistance to Enhance Prevention Capacity: a Research Synthesis of the Evidence Base
Source: Prev Sci. 2016 Feb 9;17:417–28. doi: 10.1007/s11121-016-0636-5 (PMC4839040; doi:10.1007/s11121-016-0636-5)
Supplement: Supplementary file 2 — (DOCX 75 kb) [file 11121_2016_636_MOESM2_ESM.docx]

| *Selection of Articles* | | | | |
| --- | --- | --- | --- | --- |
| Database  MEDLINE | # of Articles in Database  397 | # of Articles Kept After Review of Abstracts  76 | # of Articles Kept After De-Duplication  76 | Number of Articles Coded  6 |
| PsycInfo | 197 | 25 | 22 | 21 |
| CINAHL | 189 | 24 | 21 | 20 |
| Social Work Abstracts | 24 | 8 | 3 | 2 |
| Total | 807 | 133 | 122 | 111 |
