# Supplementary material for: Technical Assistance to Enhance Prevention Capacity: a Research Synthesis of the Evidence Base
Source: Prev Sci. 2016 Feb 9;17:417–28. doi: 10.1007/s11121-016-0636-5 (PMC4839040; doi:10.1007/s11121-016-0636-5)
Supplement: Supplementary file 3 — (DOCX 64 kb) [file 11121_2016_636_MOESM3_ESM.docx]

**The Interactive Systems Framework for Dissemination and Implementation**
