# Supplementary figures and images for: Technical Assistance to Enhance Prevention Capacity: a Research Synthesis of the Evidence Base
Source: Prev Sci. 2016 Feb 9;17:417–28. doi: 10.1007/s11121-016-0636-5 (PMC4839040; doi:10.1007/s11121-016-0636-5)

**Content Areas Addressed in Reviewed Articles:**


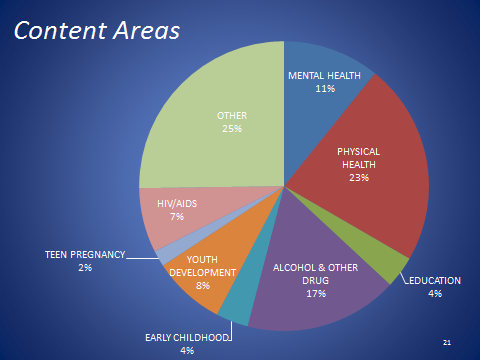

Supplement: Supplementary file 4 — (DOCX 28 kb) [file 11121_2016_636_MOESM4_ESM.docx]
