# Supplementary material for: Technical Assistance to Enhance Prevention Capacity: a Research Synthesis of the Evidence Base
Source: Prev Sci. 2016 Feb 9;17:417–28. doi: 10.1007/s11121-016-0636-5 (PMC4839040; doi:10.1007/s11121-016-0636-5)

**Coding Form**

| **Background Information** | | |  |  |  |  |  |
| --- | --- | --- | --- | --- | --- | --- | --- |
|  |  |  |  |  |  |  |  |
| Title and author(s): | | | | | | | |
|  |  |  |  |  |  |  |  |
|  |  |  |  |  |  |  |  |
|  |  |  |  |  |  |  |  |
| Content area: | | | | ☐Health | | | |
|  |  |  |  | ☐Education | | | |
|  |  |  |  | ☐Justice | | | |
|  |  |  |  | ☐Drugs and/or alcohol | | | |
|  |  |  |  | ☐Early childhood | | | |
|  |  |  |  | ☐Youth development | | | |
|  |  |  |  | ☐Teen pregnancy | | | |
|  |  |  |  | ☐After-school | | | |
|  |  |  |  | ☐Mentoring | | | |
|  |  |  |  | ☐Employment/job-training | | | |
|  |  |  |  | ☐Community development | | | |
|  |  |  |  | ☐Housing/homelessness | | | |
|  |  |  |  | ☐Other *(specify)*: | | | |
| Primary level of analysis for project: | | | | ☐Nation | | | |
|  |  |  |  | ☐State | | | |
|  |  |  |  | ☐Region | | | |
|  |  |  |  | ☐Community | | | |
|  |  |  |  | ☐Organization | | | |
| Delivery system roles represented: | | | | ☐Project director | | | |
|  |  |  |  | ☐Supervisor | | | |
|  |  |  |  | ☐Front-line provider | | | |
|  |  |  |  | ☐Other roles: | | | |

| Overall project timeline: | | | | ☐≤ one year | | | |
| --- | --- | --- | --- | --- | --- | --- | --- |
|  |  |  |  | ☐≤ two years, and > one year | | | |
|  |  |  |  | ☐≤ three years, and > two years | | | |
|  |  |  |  | ☐≤ four years, and > three years | | | |
|  |  |  |  | ☐≤ five years, and > four years | | | |
|  |  |  |  | ☐> five years | | | |
|  |  |  |  | ☐Not reported | | | |
|  |  |  |  |  |  |  |  |
|  |  |  |  |  |  |  |  |
| **Step 1: Conducting a TA Needs/Resource Assessment** | | | |  |  |  |  |
| ☐Step not reported  ☐Step reported | |  |  |  |  |  |  |
| *Information source addresses techniques associated with:* | | | | |  |  |  |
| ☐Collection of needs/resource data | | | | | | | |
| ☐Analysis of needs/resource data | | | | | | | |
| ☐Interpretation of needs/resource data | | | | | | | |
|  |  |  |  |  |  |  |  |
| Techniques for collecting needs/resource data: | | | ☐Surveys | | | | |
|  |  |  | ☐Interviews | | | | |
|  |  |  | ☐Focus groups | | | | |
|  |  |  | ☐Other *(if yes, specify)*: | | | | |
|  |  |  |  |  |  |  |  |
| Summarize any results reported from a TA needs/resource assessment: | | | | | | | |
|  |  |  |  |  |  |  |  |
|  |  |  |  |  |  |  |  |
|  |  |  |  |  |  |  |  |

| **Step 2: Establishing TA Goals and Desired Outcomes** | | | |  |  |  |  |
| --- | --- | --- | --- | --- | --- | --- | --- |
| ☐Step not reported  ☐Step reported | |  |  |  |  |  |  |
| *Information source addresses:* | | |  |  |  |  |  |
| ☐Selection of TA goal(s) | | | | ☐Selection of desired TA outcome(s) | | | |
| *If information source mentions the selection of TA goal(s):* | | | | |  |  |  |
| ☐TA goal(s) are based on a needs/resource assessment | | | | | | | |
| *If information source addresses the selection of desired TA outcome(s):* | | | | | |  |  |
| ☐Use of a systematic process (e.g., benchmarking) to select desired TA outcome(s) | | | | | | | |
|  |  |  |  |  |  |  |  |

|  |  | | |  |  | |  | |  | |  | |  | |  |
| --- | --- | --- | --- | --- | --- | --- | --- | --- | --- | --- | --- | --- | --- | --- | --- |
| **Step 3: Identifying Best TA Practices** | | | | | | |  | |  | |  | |  | |  |
| ☐Step not reported  ☐Step reported | | | |  |  | |  | |  | |  | |  | |  |
| *Information source addresses:* | | | | |  | |  | |  | |  | |  | |  |
| ☐A review process to identify TA practice(s). *If yes, see items directly below.* | | | | | | | | | | | | | | |  |
| ☐Review process informed by the available evidence-base. | | | | | | | | | | | | | | |  |
| ☐Identified candidate TA practice(s): | | | | | | | | | | | | | | |  |
|  |  | | |  |  | |  | |  | |  | |  | |  |
|  | |  |  | | |  | |  | |  | |  | |  | |
| **Step 4: Addressing Issues of Fit** | | | | | | | |  | |  | |  | |  | |
| ☐Step not reported  ☐Step reported | | | | | |  | |  | |  | |  | |  | |
| *Information source mentions:* | | | | | |  | |  | |  | |  | |  | |
| Assessing fit between TA services and TA recipients’: | | | | | | ☐Readiness for the planned TA | | | | | | | | | |
|  |  |  |  |  |  | ☐Other priorities, timelines, and deliverables | | | | | | | | | |
|  |  |  |  |  |  | ☐Daily activities and organizational operations | | | | | | | | | |
|  |  |  |  |  |  | ☐Organizational culture | | | | | | | | | |
|  |  |  |  |  |  | ☐Other existing support services and resources | | | | | | | | | |
|  | |  |  | | |  | |  | |  | |  | |  | |
|  | |  |  | | |  | |  | |  | |  | |  | |
| **Step 5: Considering Capacity Issues** | | | | | | |  | |  | |  | |  | |  |
| ☐Step not reported  ☐Step reported | | | | |  | |  | |  | |  | |  | |  |
| *Information source addresses:* | | | | |  | |  | |  | |  | |  | |  |
| Human capacities for implementing TA | | | | | ☐Internal staffing | | | | | | | | | |  |
|  |  |  |  |  | ☐External linkages, including content area specialists | | | | | | | | | |  |
|  |  |  |  |  | ☐Other human capacities *(if yes, specify)*: | | | | | | | | | |  |
| Fiscal capacities for implementing TA | | | | | ☐Funds for travel | | | | | | | | | |  |
|  |  |  |  |  | ☐Other fiscal capacities *(if yes, specify)*: | | | | | | | | | |  |
| Technical capacities for implementing TA | | | | | ☐Computer hardware | | | | | | | | | |  |
|  |  |  |  |  | ☐Software for electronic communications | | | | | | | | | |  |
|  |  |  |  |  | ☐Other technical capacities *(if yes, specify)*: | | | | | | | | | |  |
|  |  | | |  |  | |  | |  | |  | |  | |  |
|  |  | | |  |  | |  | |  | |  | |  | |  |
| **Step 6: Developing a Plan** | | | | |  | |  | |  | |  | |  | |  |
| ☐Step not reported  ☐Step reported | | | | |  | |  | |  | |  | |  | |  |
| *Information source addresses a:* | | | | | | |  | |  | |  | |  | |  |
| TA planning *process* | | | | | ☐TA planning includes collaboration between TA providers and TA recipients | | | | | | | | | |  |
| TA planning *product* | | | | | ☐TA planning product includes a timeline for activities | | | | | | | | | |  |
|  |  |  |  |  | ☐TA planning product includes roles and responsibilities for *either* TA providers *or* TA recipients | | | | | | | | | |  |
|  |  | | |  |  | |  | |  | |  | |  | |  |
|  |  | | |  |  | |  | |  | |  | |  | |  |

| **Step 7: Conducting Process Evaluation / Implementation** | | | | |  |  |  |
| --- | --- | --- | --- | --- | --- | --- | --- |
| ☐Step not reported  ☐Step reported | | |  |  |  |  |  |
| *Information source mentions:* | | |  |  |  |  |  |
| Techniques associated with generating components of process evaluation *(check all that apply)*: | | | ☐Monitored the quality of TA activities. | | | | |
|  |  |  | ☐Identified change and made midcourse corrections if needed. | | | | |
|  |  |  | ☐Tracked TA reach (e.g., attendance, participation) | | | | |
|  |  |  | ☐Assessed satisfaction with the TA process | | | | |
|  |  |  | ☐Tracked TA dosage | | | | |
|  |  |  |  |  |  |  |  |
|  |  |  |  |  |  |  |  |
| **Step 8: Conducting Outcome Evaluation** | | | |  |  |  |  |
| ☐Step not reported  ☐Step reported | | |  |  |  |  |  |
| *Information source addresses:* | | |  |  |  |  |  |
| Techniques for collecting outcome evaluation data: NA | | | ☐Surveys | | | | |
|  |  |  | ☐Interviews | | | | |
|  |  |  | ☐Focus groups | | | | |
|  |  |  | ☐Other *(if yes, specify)*: | | | | |
|  | | | | | | | |
|  |  |  |  |  |  |  |  |
| If desired (i.e., projected/planned)TA outcomes were reported (see Step 2): | | | ☐Performance on desired (Step 2) TA outcomes is either fully or partially captured in the outcome evaluation | | | | |
|  |  |  | ☐Performance on desired (Step 2) TA outcomes is not captured at all in the outcome evaluation | | | | |
|  |  |  |  |  |  |  |  |
| Summarize any results reported from a TA outcome evaluation: | | | | | | | |
|  |  |  |  |  |  |  |  |
|  |  |  |  |  |  |  |  |
|  |  |  |  |  |  |  |  |
|  |  |  |  |  |  |  |  |

| **Step 9: Continuous Quality Improvement** | | | | | | | |  | |  | |  | |  | |
| --- | --- | --- | --- | --- | --- | --- | --- | --- | --- | --- | --- | --- | --- | --- | --- |
| ☐Step not reported  ☐Step reported | | | | | |  | |  | |  | |  | |  | |
| *Information source addresses:* | | | | | |  | |  | |  | |  | |  | |
| Techniques for conducting continuous quality improvement in TA: | | | | | | ☐Techniques for continuous feedback (e.g., dashboard) | | | | | | | | | |
|  |  |  |  |  |  | ☐Quality improvement consortia /communities of practice | | | | | | | | | |
|  |  |  |  |  |  | ☐Plan-Do-Study-Act / Shewhart-based techniques | | | | | | | | | |
|  |  |  |  |  |  | ☐Other CQI techniques *(if yes, specify)*: | | | | | | | | | |
|  | |  | |  | |  | |  | |  | |  | |  | |
|  | |  | |  | |  | |  | |  | |  | |  | |
| **Step 10: Addressing Sustainability** | | | | | | |  | |  | |  | |  | |  |
| ☐Step not reported  ☐Step reported | | | | |  | |  | |  | |  | |  | |  |
| *Information source addresses:* | | | | |  | |  | |  | |  | |  | |  |
| Techniques for addressing sustainability issues in TA: | | | | | ☐Development of a sustainability plan | | | | | | | | | |  |
|  |  |  |  |  | ☐Identified a respected program champion | | | | | | | | | |  |
|  |  |  |  |  | ☐Integration of TA activities into a delivery system | | | | | | | | | |  |
|  |  |  |  |  | ☐Other sustainability techniques *(if yes, specify)*: | | | | | | | | | |  |
|  |  |  |  |  |  |  |  |  |  |  |  |  |  |  |  |
|  |  | |  | |  | |  | |  | |  | |  | |  |
|  |  | |  | |  | |  | |  | |  | |  | |  |
| **TA Relationship Features** | | | | |  | |  | |  | |  | |  | |  |
| ☐Relationship features not reported  ☐Relationship features reported | | | | | | |  | |  | |  | |  | |  |
|  | | | | |  | |  | |  | |  | |  | |  |
| TA relationship dimensions: | | | | | ☐Trust | | | | | | | | | |  |
|  |  |  |  |  | ☐Respect | | | | | | | | | |  |
|  |  |  |  |  | ☐Collaboration | | | | | | | | | |  |
|  |  |  |  |  | ☐Adjusting to readiness | | | | | | | | | |  |
|  |  |  |  |  | ☐Encouragement | | | | | | | | | |  |
|  |  |  |  |  | ☐Autonomy supportive | | | | | | | | | |  |
|  |  |  |  |  | ☐Shared expectations about roles | | | | | | | | | |  |
|  |  |  |  |  | ☐Building rapport | | | | | | | | | |  |
|  |  |  |  |  | ☐Other relationship dimensions *(if yes, specify)*: | | | | | | | | | |  |
|  |  |  |  |  |  |  |  |  |  |  |  |  |  |  |  |
|  |  |  |  |  |  |  |  |  |  |  |  |  |  |  |  |
|  |  | |  | |  | |  | |  | |  | |  | |  |
|  |  | |  | |  | |  | |  | |  | |  | |  |

| **Implementation Life Span Stages Targeted by TA** | | |  |  |  |  |
| --- | --- | --- | --- | --- | --- | --- |
| ☐Content not reported | | |  |  |  |  |
| ☐Content reported |  |  |  |  |  |  |
| *Stage in Life Span* |  |  |  |  |  |  |
| ☐Initiation of an innovation (occurs prior to implementing an innovation) | | | | | | |
| ☐Implementation of an innovation (work involved in bringing an innovation into practice) | | | | | | |
| ☐Stability of an innovation (subsequent to implementation, refers to efforts around sustaining an innovation ) | | | | | | |

**Codebook**

**
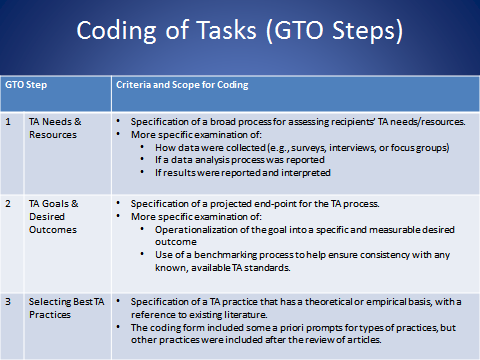
**

**
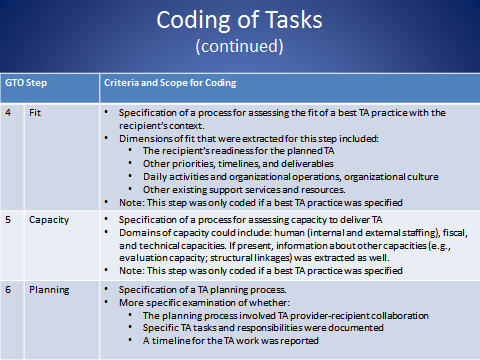
**

**
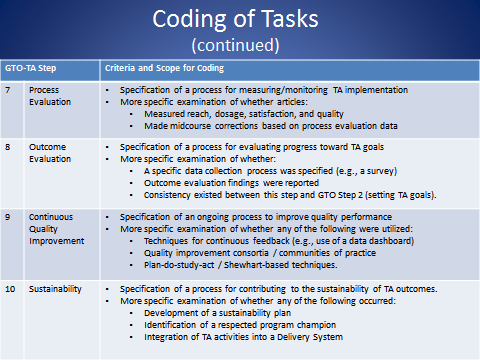
**


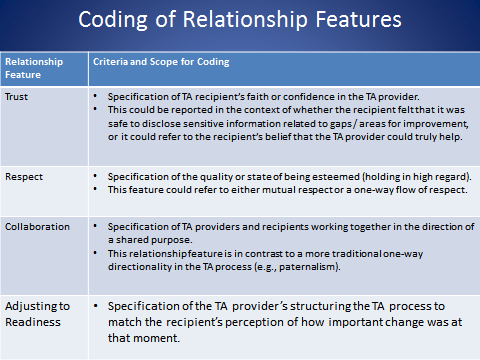


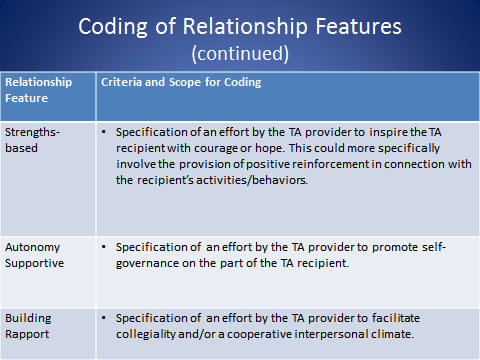


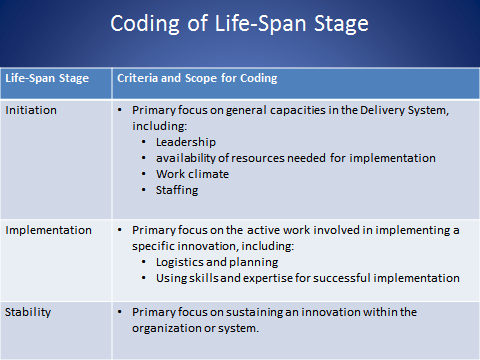

Supplement: Supplementary file 5 — (DOCX 13 kb) [file 11121_2016_636_MOESM5_ESM.docx]
